# Development and characterization of hydroxyapatite and multiwall carbon nanotubes reinforced polypropylene biocomposites

**DOI:** 10.1038/s41598-025-96082-8

**Published:** 2025-05-28

**Authors:** Abdel Rahman Elmofty, Marwa E. Abdel Aziz, Mahmoud Tash, Shimaa El-Hadad

**Affiliations:** 1 Mining, Petroleum and Metallurgical Engineering Department, Faculty of Engineering, Cairo University, Giza, Egypt; 2 Central Metallurgical Research and Development Institute, Helwan, Cairo, 11722 Egypt

**Keywords:** Polypropylene nanocomposites, Nano-hydroxyapatite, Multiwall carbon nanotubes, Biocompatibility, Biomaterials, Polymers

## Abstract

Two groups of Polypropylene/hydroxyapatite nanocomposites (PP/nHA) were fabricated by melt blending of PP with (5, 10, and 20 wt%) of hydroxyapatite nanoparticles nHA with two different sizes (~ 40 and 90 nm). The nanocomposites were prepared without and with the addition of 0.3 wt% of multiwall carbon nanotubes (MWCNTs). The effect of HA size and content as well as the addition of MWCNTs on the structure, thermal and mechanical properties of the prepared composites was investigated. All the samples were immersed in simulated body fluid (SBF) to compare their ability to precipitate HA from SBF. Scanning electron microscope (SEM), X-ray diffraction analysis (XRD), thermogravimetric analysis (TGA), and tensile strength tests were used for the investigation. XRD patterns reveal that incorporating nHA particles and/or MWCNT into the polymer matrix does not cause any structural change to the PP crystalline lattice. SEM shows a significant improvement in the dispersion of nHA particles along PP matrix with a smoother surface morphology by the addition of MWCNTs. Based on the thermal studies, both nHA particles and MWCNTS were known to enhance the thermal stability of the prepared composites. The tensile strength of PP increased by 20% for PP-5% (90 nm) HA-MWCNTs composites and 44% for PP-5% (40 nm) HA-MWCNTs composites. Furthermore, in vitro studies reveal the apatite-forming ability on the surface of the prepared composites especially those prepared using MWCNTs.

**Supplementary Information:**

The online version contains supplementary material available at 10.1038/s41598-025-96082-8.

## Introduction

Biomaterials such as metallic alloys, ceramics, polymers, and composites have been progressively developed and used as medical implants^1,2^. Recently, many bone diseases have arisen due to the vast use of technology^3^. It was reported that the use of biomaterials in orthopedic applications has increased by 40% from 2015 to 2020, and it is expected to reach up to 58 billion by 2027^4,5^. To be used in human bodies they should exhibit a combination of properties such as high mechanical properties, affordability, biocompatibility, decreased risk of infection and inflammation, and osteo-regeneration^6^. Although metals are the most commonly used biomaterials for fracture fixation, metallic biomaterials still face challenging limitations, such as the inflammation that may happen due to the release of undesired metallic ions in a highly reactive environment^7^. Moreover, the high elastic modulus of the metallic implants relative to that of natural bones leads to stress shielding, which addresses more challenges^1,2^. On the other hand, polymer bio-composites, are recently the most researched materials for tissue replacements due to variety of reasons such as their relative biocompatibility, similarity in composition and structure to natural tissue, and simplicity of production^8,9^. They can be developed to fulfill a range of specialized tasks, depending on the targeted application^10–16^. Furthermore, the utilization of polymer-based bio-composites in medical applications requires additional improvements, where several challenges have to be overcome.

One of the worth studying polymers is PP due to its low cost, transparency, dimensional stability, recyclability, and natural flame resistibility. Moreover, PP has low toxicity, excellent electrical insulation, and low moisture absorption and it is also easy to sterilize^17,18^. However, its superiority over other polymers used in biomedical industries is due to its capacity to be autoclaved.

Hydroxyapatite (HA) is a ceramic material that can be considered as one of the most important reinforcing phases, as is close in its chemical composition to the human bone, and is well-known for its osteo-inductive-ability, biocompatibility, and degradability. As a result, it is used extensively as a tissue engineering material^19–24^. However, HA faces some limitations such as its low mechanical properties compared with the bone^25^. To overcome HA drawbacks, several polymer/ceramic HA composites were developed with excellent mechanical characteristics and biocompatibility as well^23,26,27^. Recently, researchers worldwide started to utilize different carbonaceous materials reinforcements such as carbon nanotubes^28–32^ including single-wall (SWCNTs), double-wall (DWCNTs), and multiwall (MWCNTs)^33,34^, to fabricate composite materials with functional properties^35–39^.

Jiao et al.^40^ have reported that Polycarbolactone (PCL) reinforced with 20 wt% of nano HA particles showed tensile strength and flexural strength higher than that of PCL reinforced with the same weight percentage of micro-HA particles. Zebarjad et al.^41^ observed that, adding more than 2.5 wt% of HA nanoparticles to Polymethylmethacrylate (PMMA) matrix decreased both the yield and the ultimate compression strength. Wear rate was found to decreases by increasing HA content in both the atmosphere and artificial saliva. Liao et al.^28^ reinforced polypropylene with 0.1 and 0.3 wt% of MWCNTs and 8, 15, and 20 wt% of nHA nanorods to fabricate bio-composites. Testing of these reinforced PP demonstrated that the MWCNTs addition is beneficial in enhancing the stiffness, tensile strength, and impact toughness of the PP/nHA nanocomposites. In the recent review of Maghima et al.^42^, it was concluded that polymer composites reinforced with carbon nano-tubes are vital for shaping the future of biomedical technology. This comes from their mechanical properties and biocompatibility which make them suitable choices for biomedical devices. In addition to their ability to enhance cellular proliferation and adhesion, that makes them attractive for tissue-engineering purposes. Adding two different reinforcements at the same time while processing PP, may expand the application field of PP composites by combining their properties as reviewed by Maria and Caren^43^.

From the aforementioned studies, it is obvious that polymer-based composites fabricated using HA particles are promising biomaterials. Especially in the low load-bearing applications such as maxillofacial plates. However, there is a lack of research on the effect of HA particle size and the addition of MWCNTs on the mechanical properties and biocompatibility of polymer composites. Accordingly, the present work has a general objective of providing a new approach to repair damaged bones by synthesis and development of novel biomaterials, such as polymeric composites that will be of great aid in bone tissue engineering and regeneration. In particular, two series of polymer composites were synthesized using two different sizes of hydroxyapatite nanoparticles together with fixed MWCNTs content by melt blending in a twin screw extruder instrument. The influence of different sizes and contents of HA nanoparticles on the structure, mechanical, thermal and invitro cell responses of the produced polymer composites was studied. Furthermore, the effect of incorporating MWCNTs as reinforcement on the properties of the produced polymer composites was evaluated.

## Experimental work

### Materials

Polypropylene AV161 block copolymer pellets with density of 0.9 g/cm^3^, melt flow rate of 5.5 g/10 min, and melting temperature range of 190–230 °C were obtained from Petro Rabigh (Saudi Arabia). Hydroxyapatite nano particles with two different sizes of around 40 nm and 90 nm were purchased from Unionchem Enterprise Corp (China), multiwall carbon nanotubes with diameters of 15 ± 7 nm, purity of 94.5% were supplied by Nanotech Egypt for Photo Electronics.

### Preparation of nanocomposites

**Fig. 1:**
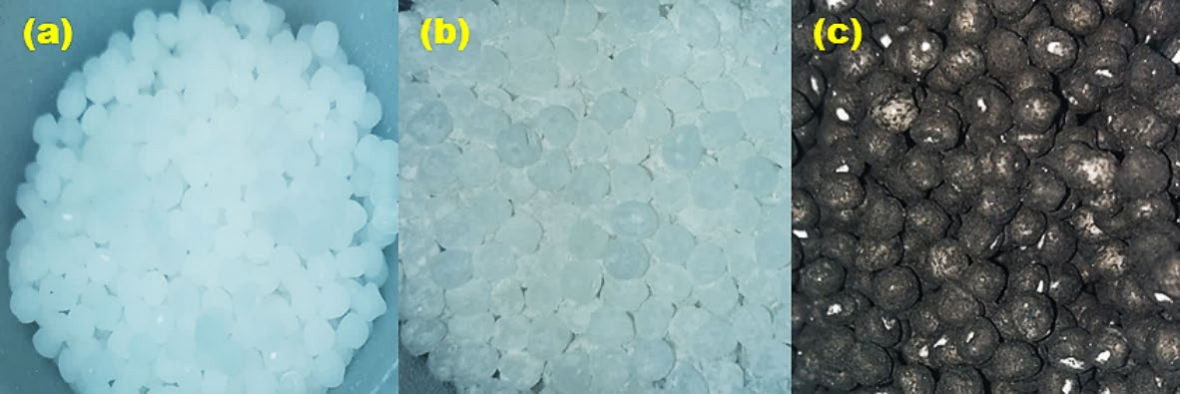
PP pellets covered with thin film of oil paraffin (**a**), PP pellets mixed with nHA powder (**b**) and PP pellets mixed with nHA and MWCNT powder (**c**).

Two groups of samples (PP/nHA) were prepared by melt blending of PP with three different weight percents (5, 10 and 20%) of two different sizes (~ 40 and 90 nm) of nHA. The nanocomposites were prepared with the addition of 0.3 wt.% MWCNT and without, as shown in Table 1. Samples prepared using HA nanoparticles with particle size around 40 nm were referred to as group A, and those prepared from HA nanoparticles with particle size around 90 nm were referred to as group B. The weight of each sample was about 30 g with the proportions listed in Table 1. To prepare the nanocomposites, few drops of oil paraffin were added to PP pellets to ensure that each pellet is covered with a thin film of oil paraffin as shown in Fig. 1a, which facilitates the adhesion of nano powder to the polymer pellet, then half of the amount of nHA or (nHA + MWCNT) powders was added to the pellets, and mixed using a high-speed mixer at about 120 rpm for 3 min, after that the remaining of the nano powder was added and mixed again for another 2 min. The resulting pellets are shown in Fig. 1b and c.

**Table 1:** Composition (wt.%) of group A samples (samples prepared with around 40 nm HA) (a) and composition of group B samples (samples prepared with around 90 nm HA) (b).

Sample no.	Sample name	PP (wt.%)	HA (wt.%)	MWCNT (wt.%)
(a) Group A samples				
A1	PP-5% HA	95% (28.5 gm)	5% (1.5 gm)	–
A2	PP-10% HA	90% (27 gm)	10% (3 gm)	–
A3	PP-20% HA	80% (24 gm)	20% (6 gm)	–
A4	PP-5% HA-0.3% MWCNT	94.7% (28.41 gm)	5% (1.5 gm)	0.3% (0.09 gm)
A5	PP-10% HA-0.3% MWCNT	89.7% (26.91 gm)	10% (3 gm)	0.3% (0.09 gm)
A6	PP-20% HA-0.3% MWCNT	79.7% (23.91 gm)	20% (6 gm)	0.3% (0.09 gm)
(b) Group B samples				
B1	PP-5%HA	95% (28.5 gm)	5% (1.5 gm)	–
B2	PP-10%HA	90% (27 gm)	10% (3 gm)	–
B3	PP-20%HA	80% (24 gm)	20% (6 gm)	–
B4	PP-5%HA-0.3% MWCNT	94.7% (28.41 gm)	5% (1.5 gm)	0.3% (0.09 gm)
B5	PP-10%HA-0.3%MWCNT	89.7% (26.91 gm)	10% (3 gm)	0.3% (0.09 gm)
B6	PP-20% HA-0.3% MWCNT	79.7% (23.91 gm)	20% (6 gm)	0.3% (0.09 gm)

**Fig. 2:**
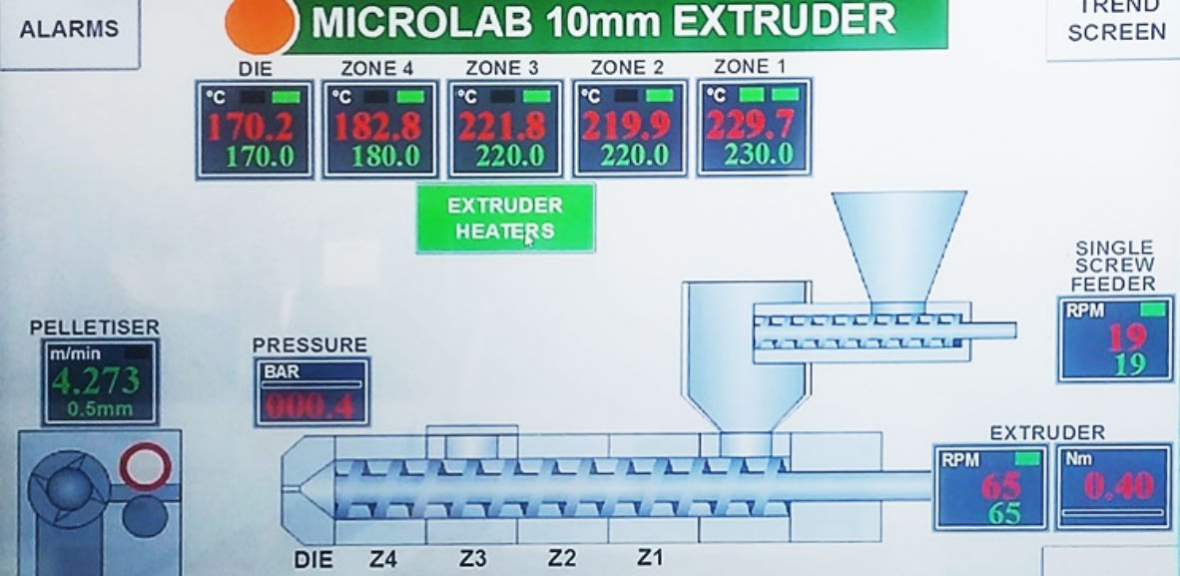
Heating temperatures of the five-barrel zones of the extruder form the hopper to the die.

**Fig. 3:**
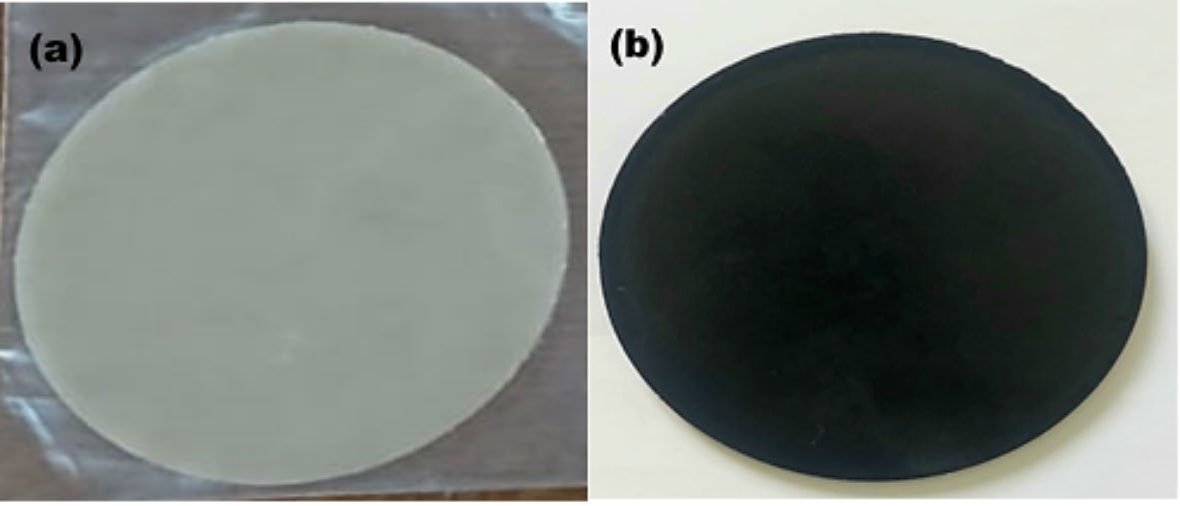
PP/nHA compressed disc (**a**) and PP/nHA/MWCNT compressed disc (**b**).

After mixing, the components were double extruded using 10 mm Rondol twin screw extruder with a single screw volumetric main feeder, where the single screw feeder was adjusted at 19 rpm, the extruder was set at 65 rpm, and the rate of the pelletizer was 4.273 m/min. The blending temperatures of the five-barrel zones of the extruder from the hopper to the die were maintained at 230–220–220–180–170 °C, respectively as shown in Fig. 2. After extrusion the resulting filaments were cut to 0.5 mm pellets using the pelletizer. The pellets of each sample were dried in an oven at a temperature of 70 °C for 24 h, then hot pressed using 10 ton manually operated bench top hydraulic press (C3422) at a temperature of 210 °C to form discs of 100 mm in diameter and 3 mm in thickness for the purpose of characterization as shown in Fig. 3a and b. The initially applied pressure was set at 40 MPa for 5 min, then the pressure was increased to 75 MPa for another 5 min. Finally, the discs were left for free cooling to room temperature.

### Characterization

#### Structure

X-ray diffraction analyses were carried out on some of the prepared nanocomposite samples to identify different phases in the produced bio composite samples, as well as the effect of both nHA particles and MWCNTs on the crystalline structure of PP polymer. A refractometer “Siemens D500” equipped with Cu-K_α_ radiation, and operated at 40 kV and 30 mA. The data were collected from 10 to 40°at a scanning rate of 0.02°/s. The structure of the prepared nanocomposite samples was investigated using SEM (Thermoscientific Quattro S). The samples (about 1 mg) were coated with a thin layer of chromium using vacuum sputter coater DST1 Vactechniche prior to the SEM investigation.

#### Thermal analysis

Thermo gravimetric analysis (TGA) of PP and the prepared nanocomposites (about 1 mg of each sample) were carried out using a TGA Instrument (TGA Q50) under nitrogen atmosphere at a heating rate of 10 °C/min. The weight percentage change of the specimens from 30 to 600 °C was recorded. Both the degradation temperatures at 5% and 50% weight loss were determined and referred to as the initial decomposition temperature (T_i_) and the temperature at which half of the material decomposes (T_1/2_).

#### Mechanical characteristics

**Fig. 4:**
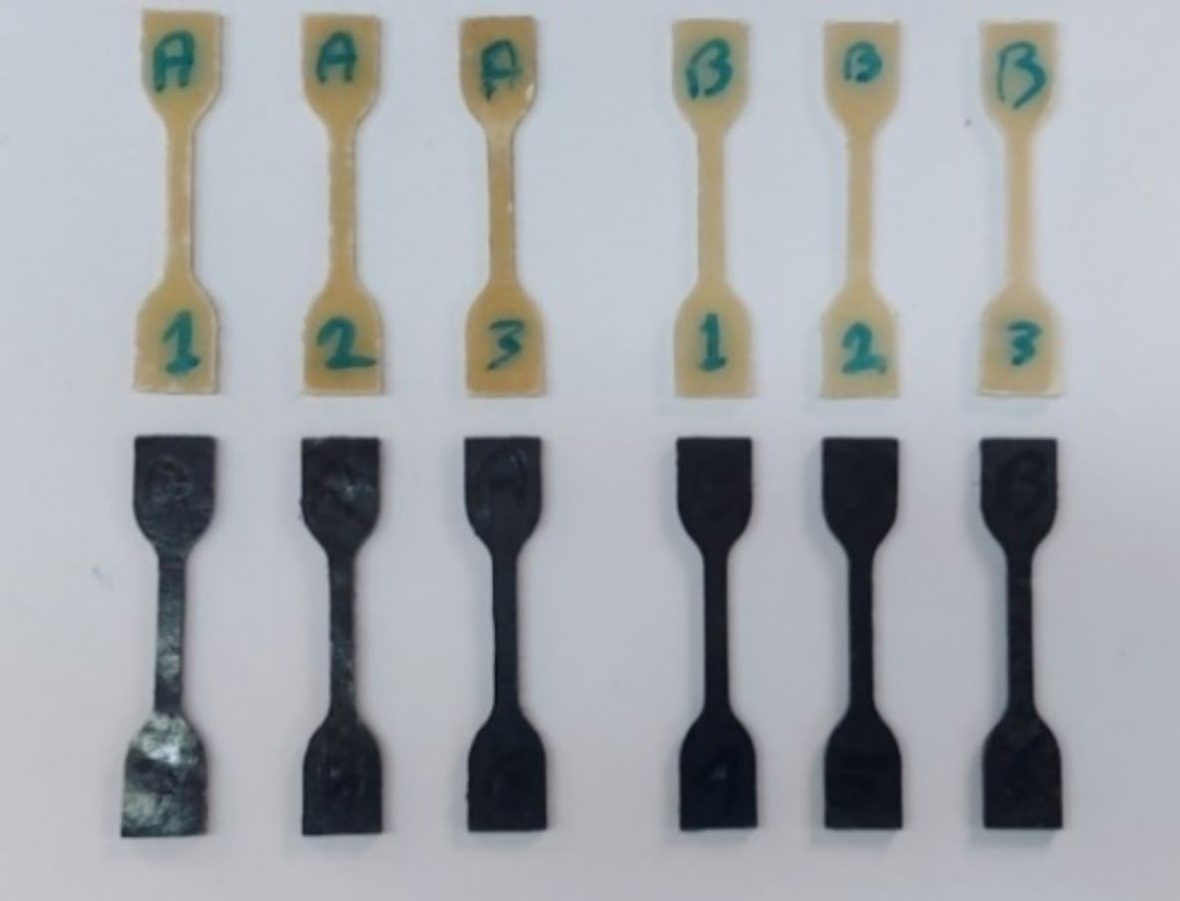
Shape of TSC ISO 527–2-5A dumb-bell shaped tensile bars.

The hot-pressed sheets were cut into dumb-bell shaped tensile bars, sizing as per TSC ISO 527-2-5A using Ray Ran Pneumatically Operated Test Sample Cutting Press as shown in Fig. 4. Tensile tests were carried out using a Shimadzu Autograph AGS tester, following ISO 527 Standard, with a crosshead speed of 50 mm/min at room temperature and relative humidity of 50%. At least three specimens of each composition were examined, and the average values were taken.

#### Hardness testing

Vickers hardness of the prepared nanocomposite samples was measured using a Zwick Roell hardness tester according to ASTM D2240. Rectangular specimens with the dimensions of 2 × 3 cm and a thickness of 3 mm were cut from the hot-pressed sheets. The specimens were mounted in an acrylic resin, then polished using silicon carbide papers to obtain scratch-free surfaces. An indentation load of 500 g was applied and each sample was indented for at least 5 times at the center.

#### In vitro compatibility

In vitro evaluation for apatite-forming ability of implant materials was carried out on some of the prepared nanocomposite samples (A1, A4, B1, B4), following ISO 23317 standard. The samples were cut into 10 mm diameter discs with a thickness of 3 mm and then immersed in the simulated body fluid that was prepared according to reference^44^. All samples were immersed in 10 mL of SBF and placed in an incubator. The immersion took place at a temperature of 36.5 °C for 30 days. After 30 days, the samples were removed from the incubator, washed gently with deionized water, then placed in an oven at a temperature of 100 °C for 3 h to be dried^45^. After that, the surface morphology of the samples was studied using SEM (Thermoscientific Quattro S). The samples were coated with a thin layer of Chromium using a vacuum sputter coater DST1 Vactechniche before the SEM examination. The chemical composition of the same selected samples after immersion in SBF was measured using the high energy dispersive X-ray instrument (EDX; Ametek Z2e analyzer–Z240) attached to the SEM instrument. The bioactivity of the material was evaluated by confirmation of apatite formation on the surface of the material.

## Results and discussion

### X-ray diffraction analysis

**Fig. 5:**
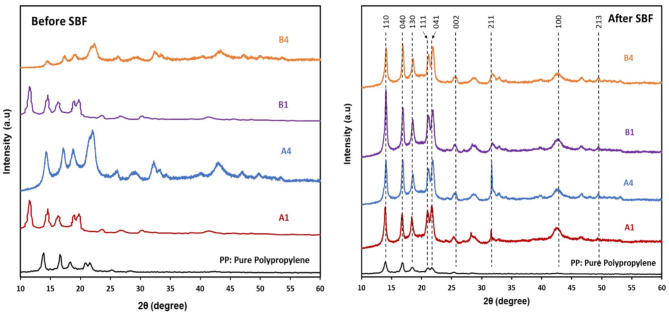
Typical XRD patterns of PP and nanocomposite samples; before (**a**) and after immersion in SBF (**b**).

XRD patterns of PP and PP/nHA nanocomposites with and without the addition of MWCNT before and after immersion in SBF are shown in Fig. 5a and b, respectively. For PP, the characteristic peaks of the pattern are 2θ = 13.88, 16.92, 18.84, 21.4 and 22.04°, corresponding to the (110), (040), (130), (111) and (041) planes of the α-PP crystals, respectively. However, new diffraction peaks appear at 2θ = 31.77 and 49° corresponding to the (211) and (213) planes of the nanocomposites spectra which are the characteristics of nHA particles, and they differ slightly from one sample to another. Another new diffraction peaks appear at 2θ = 25 and 43.2° corresponding to the (002) and (100) planes and are characteristics of MWCNTs. It can be seen from the XRD patterns that all PP-nHA nanocomposites both with and without the addition of MWCNT have nearly the same diffraction peaks as that of Pristine PP, indicating that the incorporation of nHA particles and/or MWCNT into the polymer matrix does not cause any structural change to the PP crystalline lattice^27,28^. After immersion in SBF, all the samples exhibited the same diffraction peaks, the only observable change is that the peaks became sharper and more intense, which may be attributed to the formation of a crystalline layer on all the samples.

### Microstructures

**Fig. 6:**
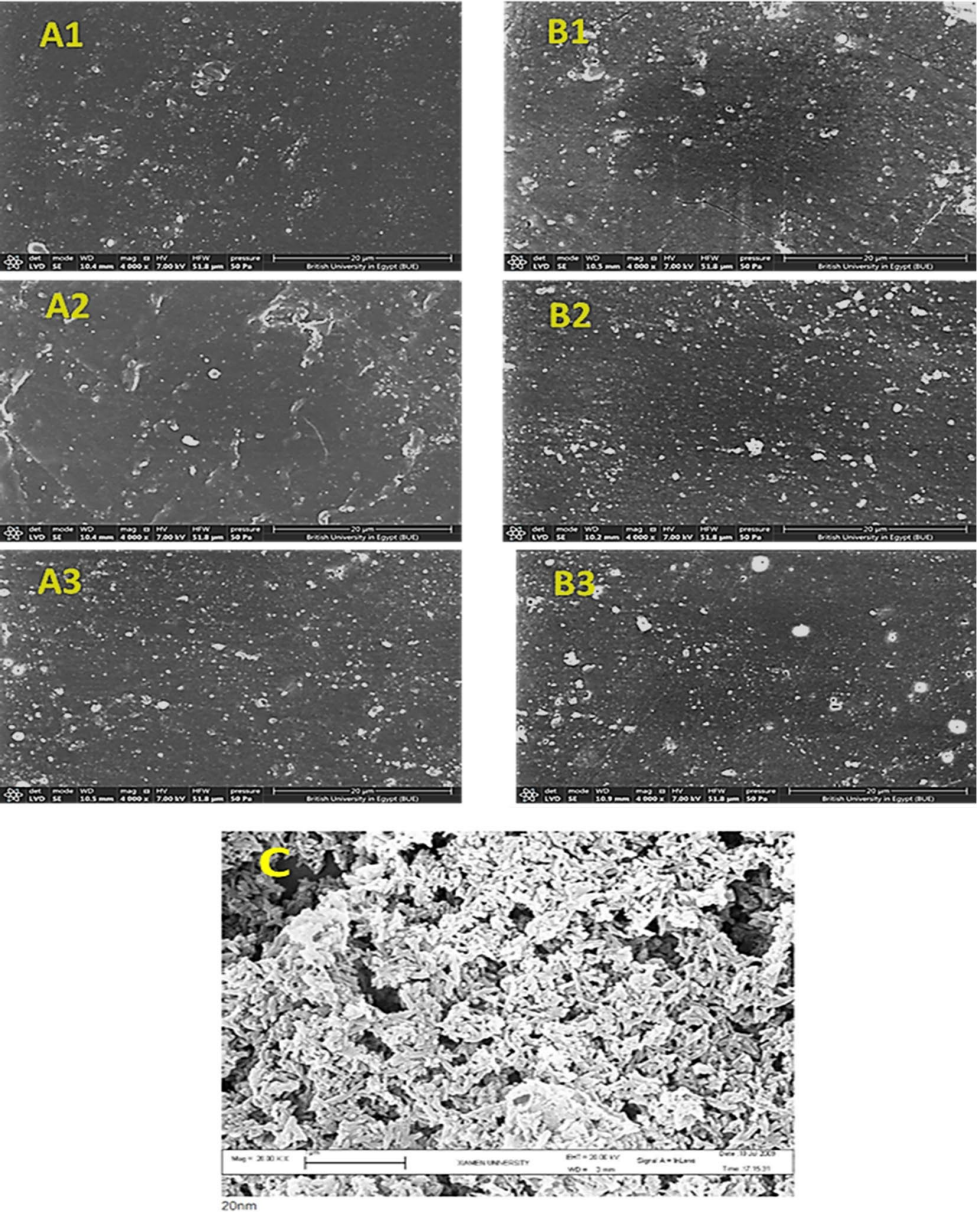
SEM micrographs of the PP nanocomposite samples; (**A1**–**A3**) and (**B1**–**B3**) and (**C**) showing needle-shaped hydroxyapatite nanoparticles.

**Fig. 7:**
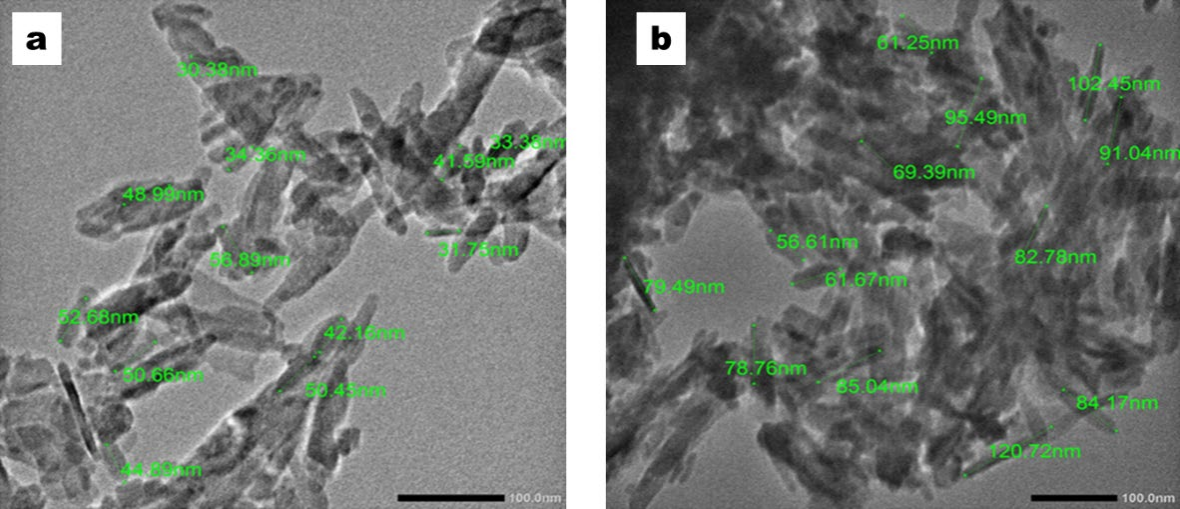
TEM images (**a**) ~ 40nm hydroxyapatite nanoparticles and (**b**) ~ 90 nm hydroxyapatite nanoparticles.

SEM micrographs of the prepared PP nanocomposites containing 5, 10 and 15 wt.% of HA nanoparticles without MWCNTs (A1, A2, A3 samples prepared using nHA particles of size around 40nm and B1, B2, B3 samples having nHA particles of size around 90 nm) together with the micrographs of nHA particles as provided from the manufacturer are shown in Fig. 6.  The micrographs of A1and B1 samples containing 5 wt% nHA particles without MWCNTs exhibit a rough surface pattern with many nHA particles randomly dispersed within the PP matrix, appearing as white bright dots, stacking on the PP matrix dark surface due to the poor biocompatibility between the PP bioinert surface and nHA bioactive surface in the absence of MWCNTs. By increasing the content of nHA particles up to 10 and 15 wt% nHA particles were dispersed in the form of small and large aggregates within the PP matrix as shown in the micrographs of A2, A3, B2, B3 samples. SEM micrograph of the needle-shaped nHA particles can be seen in Fig. 6 C. It can be seen also that the SEM micrographs of group A are nearly the same as that of group B except for a few larger particle aggregations, which means that the particle size of HA nanoparticles has a minimum effect on the microstructure of the prepared composites. A sample of about 1mg of each size of hydroxyapatite nanoparticles was dispersed in 2 ml ethanol, then imaged using TEM (JEM-2100PLUS) instrument and the resulted images are seen in Fig. 7.

It can be seen from Fig. 7 that the average particles size of the first sample of hydroxyapatite nanoparticles (sample a) is around 40 nm, while the average particles size of the second sample of hydroxyapatite nanoparticles (sample b) is around 90 nm.

**Fig. 8:**
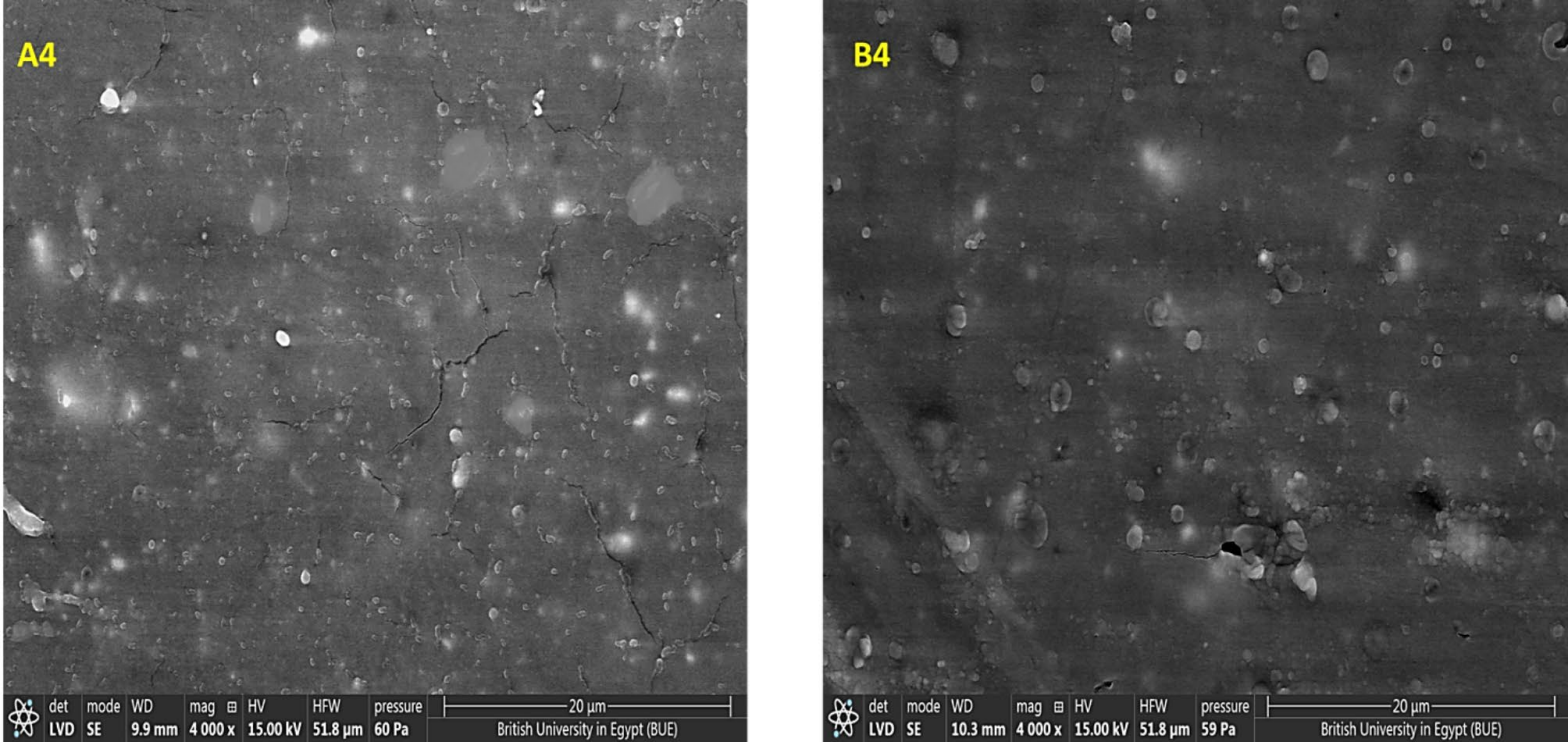
SEM micrographs of the PP nanocomposite with the addition of MWCNTs; (**A4**) (PP-5 wt% HA-0.3 wt%MWCNTs) and (**B4**) (PP-5 wt%HA-0.3 wt%MWCNTs).

However, the addition of MWCNTs caused a significant improvement in the dispersion of nHA particles along PP matrix with a smoother surface for both group A and group B samples, as nHA particles are homogeneously distributed within the PP matrix with the presence of small number of aggregated nanoparticles as shown in Fig. 8A4, B4, so it can be concluded that the addition of MWCNTs enhances the biocompatibility of the prepared nanocomposites.

### Thermal analysis

**Fig. 9:**
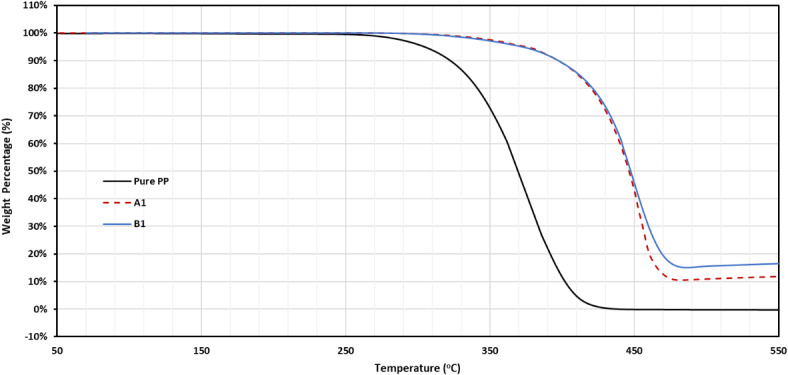
TGA curves of PP, A1, and B1, similar curves for the other composites can be found in the supplementary Fig. S1.

TGA studies were carried out on the nanocomposite samples where the difference in the weight of the sample was determined upon heating from 50 to 550 °C as a function of temperature as shown in Fig. 9. As evident, the thermograms of the nanocomposites for both groups A and B, without and with the addition of MWCNTs exhibit the same profile as the thermal degradation take place in one step. The values of the initial decomposition temperature (T_i_) and the temperature at which half of the material decomposes (T_50_) of the investigated samples are given in Table 2. It can be noted that there is an improvement in the thermal stability of PP matrix with the addition of the nHA particles in both groups and the improvement is more in the presence of MWCNTs, which is indicated by the shift of T_i_ and T_50_ to higher values.

**Table 2:** The values of the initial decomposition temperature (T_i_) and the temperature at which half of the material decomposes (T_50_) of PP and the investigated nanocomposite.

Sample	T_i_ (^°^C)	T_50_(^°^C)
PP	281	385
A1	334	447
A2	357	454
A3	351	450
A4	377	454
A5	342	455
A6	360	457
B1	312	447
B2	315	450
B3	302	446
B4	334	449
B5	339	455
B6	336	449

From Fig. 9 and Table 2, it can be observed that the values of T_i_ and T_1/2_ increase by increasing the amount of nHA particles as shown in the curves of A1 and A2 samples (having 5 wt.% and 10 wt.% nHA respectively) as the addition of nHA particles would somewhat limit the mobility of polymeric chains through chemical or physical interfacial bonds, which increases the energy needed for decomposition and explains the higher thermal stability of PP/nHA composites when compared to neat polymers^46^. By increasing the amount of nHA up to 15 wt.%, the values of T_i_ and T_1/2_ show a slight decrease, as HA particles will be agglomerated, which causes irregularity in some regions of the prepared composites, these regions were easier to be thermally decomposed, leading to a decrease in the thermal stability. Also, it can be seen that the addition of MWCNTs causes significant enhancement in the thermal stability as indicated by the shift of T_i_ and T_1/2_ values from 334^⁰^C and 447^⁰^C (sample A1) to 377 °C and 454 °C (sample A4), which ensures that MWCNTs is more effective in the improvement of the thermal stabilities and hence the compatibility of the prepared composites at low content of nHA (5 wt.%). Nearly the same behavior is observed for group B samples (90 nm HA particles), indicating that the size of nHA particles doesn’t have a clear impact on the thermal stabilities of the prepared composites.

### Tensile strength

**Fig. 10:**
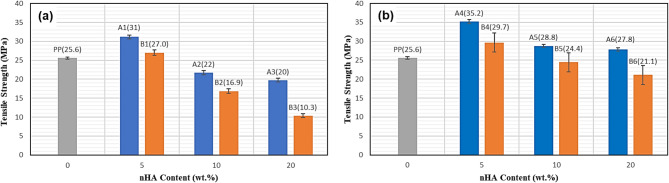
Tensile strength with nHA particles content (wt.%); PP/nHA nanocomposites (**a**) and PP/nHA/MWCNT nanocomposites (**b**).

Figure 10a and b show the ultimate tensile strength as function of the content of the nHA particles in the nanocomposites (PP/nHA and PP/nHA/MWCNT) together with the ultimate tensile strength of pure PP polymer. It can be seen from Fig. 10a that the ultimate tensile strength of PP is 25.6 MPa, which increases up to 31 MPa by the addition of 5 wt.% nHA particles with size of 40 nm (sample A1) and increases up to 27 MPa by the addition of 5 wt.% nHA particles with size of 90 nm (sample B1). But the ultimate tensile strength decreases slightly with further increase of nHA particles up to 10 and 15 wt.% nHA for both sizes. The same finding is reported by Liu and Wang who prepared PP/HA composites reinforced with 10, 20 and 25 vol. % HA microparticles^47^. It was found that the ultimate tensile strength of PP decreases by adding 10, 20 and 25 vol. % HA. The reduction in the tensile strength may be attributed to the agglomeration of nHA particles with contents higher than 5 wt.%. Figure 10b shows that the tensile strength of the nanocomposites is significantly enhanced by the addition of low percent of MWCNTs (0.3 wt.%) where the tensile strength of PP increases from 25 up to 35.2 MPa (sample A4) and 30 MPa (sample B4). It is clearly observed that the values of strength are higher for group A samples with smaller size of nHA particles (40 nm) than group B samples with greater size of nHA (90 nm).

**Fig. 11:**
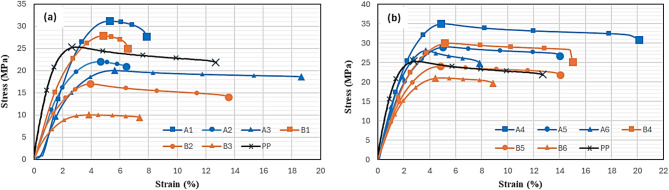
Stress- Strain curves for PP/nHA nanocomposites (**a**) and PP/nHA/MWCNT nanocomposites (**b**).

Typical tensile stress–strain curves of the prepared nanocomposite samples are shown in Fig. 11.

With an early linear section that defines the slope that decreases after a specific strain level and indicates the material stiffness that relaxes as the failure load approaches, all curves exhibit roughly similar behavior. Within each group (a) or (b), it can be seen that the values of the strength decrease with increasing nHA content which may be attributed to the formation of aggregates that act as mechanical failure concentrators. By comparing the strength of group (a) samples with that of group (b) samples, it can be seen that the values of strength are higher for group (b) samples which might be attributed to the efficient stress transfer from the polymer matrix to the stiff MWCNTs through the interface. Table 3 compares the Mechanical properties of some of the prepared PP/HA composites to other PP/HA prepared in literature and the cortical bone.

**Table 3:** Comparison of Mechanical properties of some of the prepared PP/HA composites to other PP/HA prepared in literature and cortical bone.

Material	UTS (MPa)	References
Cortical bone	126.3 $\pm$ 33.1	^39^
10%HA–PP	26.32	^47^
30 wt% HA-PEEK	~ 55	^48^
20%nHA-PP-0.3%MWCNTs	31	^27^
20%nHA-PP-0.3%MWCNTs	31	^28^
Pure PP	25.6	–
A1	31	–
A4	35.2	–
B1	27	–
B4	29.7	–

### Hardness testing

**Fig. 12:**
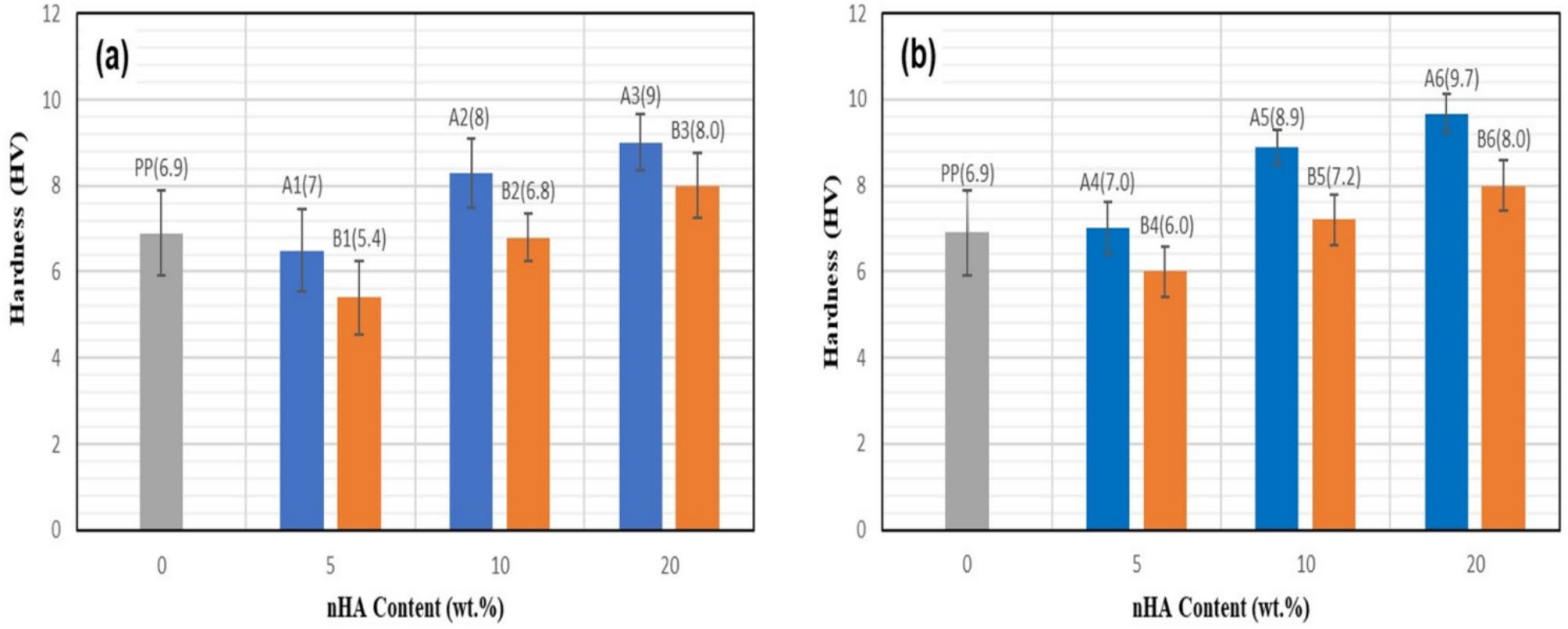
Hardness results with nHA particles content (wt.%); PP polymer, PP/nHA nanocomposites (**a**) and PP/nHA/MWCNT nanocomposites (**b**).

The values of the hardness for pure PP polymer and the prepared composite samples are shown in Fig. 12. The hardness values increase with an increase in nHA particles content, and for each sample the hardness is enhanced with MWCNT addition. Some previous researches reported the relationship between MWCNT content and hardness values for HDPE/HA composites^49^. In the present work, the effect of nHA particles size and content as well as the presence of fixed MWCNT percent were studied.

**Fig. 13:**
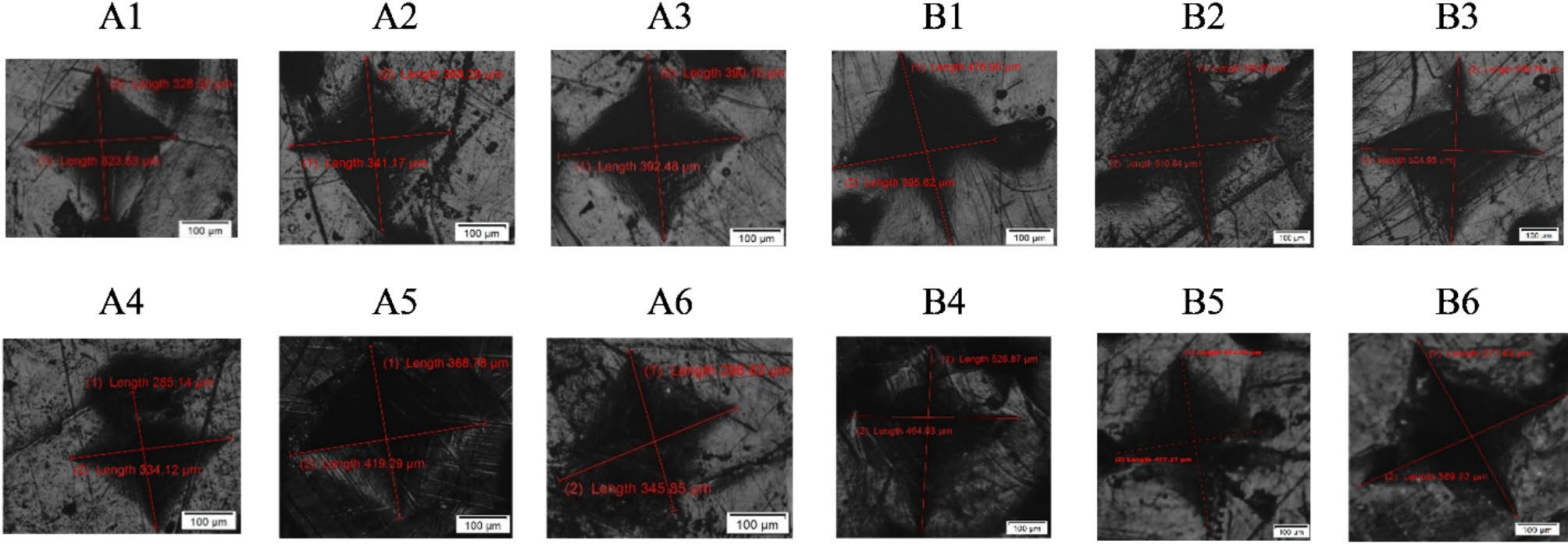
Surface morphology after indentation.

It can be seen from Fig. 12 that the values of hardness are higher for group A samples, indicating that the smaller size of nHA particles has a more enhanced effect on the hardness properties than the larger size. Figure 13 shows the surface morphology after deformation by the press indentation, where B samples exhibited distorted indentations. The above results show that the prepared nanocomposite samples have a promising mechanical property, and can be investigated further for evaluating the apatite-forming ability.

### In vitro evaluation of apatite-forming ability of implant materials

**Fig. 14:**
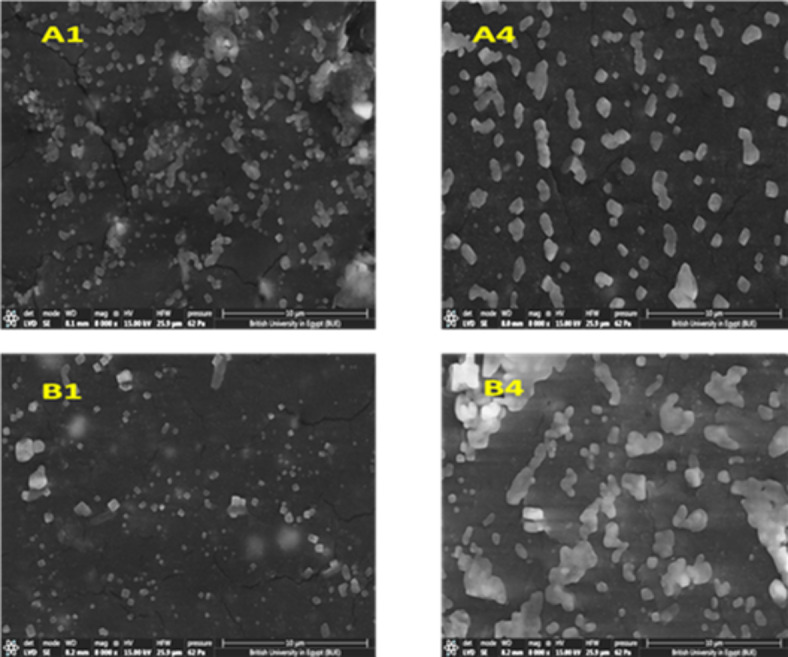
SEM micrographs of the PP based nanocomposite samples (A1, A4, B1 and B4) at magnification of 8000 × after immersion in SBF for 30 days.

**Fig. 15:**
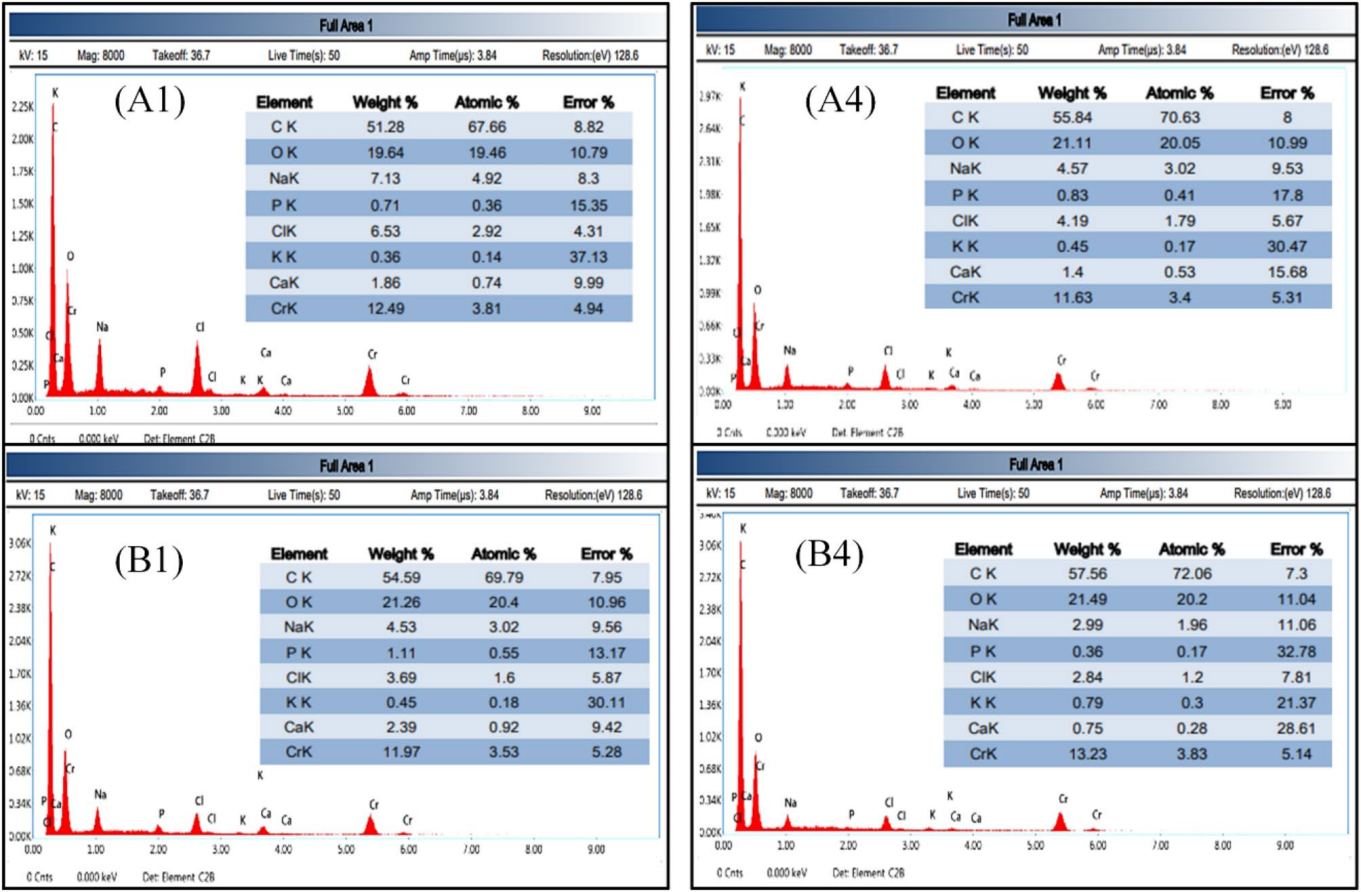
EDX for samples (**A1**, **A4**, **B1**, and **B4**) after immersion in the SBF.

Figure 14 shows SEM micrographs of samples A1, A4, B1 and B4 after immersion in SBF for 30 days. EDX analysis of A1, A4, B1, and B4 showing Ca and P peaks after immersion is shown in Fig. 15. By comparing Figs. 8 and 14, it can be seen that there are round-shaped deposits on the surface of the four samples after immersion in SBF, indicating the formation of bone-like hydroxy apatite layer mainly from Calcium and Phosphorous^50,51^. A comparison between the micrographs of samples A1 and B1 (without MWCNTs) with those of A4 and B4 (reinforced with MWCNTs) signifies that nanocomposite reinforced with 0.3 wt.% MWCNTs has a negligible effect on the precipitation rate of apatite on the polymeric composite surface. Also, it is clear that the microstructures of the nanocomposite samples reinforced with 40 nHA particles (A1and A4) show more salts precipitation on the surface than the nanocomposite samples reinforced with 90 nHA particles (B1 and B4).

The ratios of Ca/P obtained from EDX analysis of Fig. 15 were summarized in Table 4. The results show that all the samples are composed of carbon, oxygen, phosphate, calcium, and chromium. Before immersion in SBF, Ca/P ratios were 1.74, 2.08, 2.15 and 1.69 for samples A1, A4, B1 and B4, respectively. However, after immersion, the Ca/P ratios are 2.64, 2.19, 2.17 and 2.02 for the same samples with the same order. These results confirmed the apatite-forming ability for all the prepared nanocomposite samples after immersion in SBF and the apatite forming ability is higher for the nanocomposite samples reinforced with smaller size of nHA particles (40 nm).

**Table 4:** Ca and P wt% for samples A1, A4, B1 and B4 before and after immersion of SBF.

	Before immersion				After immersion			
	A1	A4	B1	B4	A1	A4	B1	B4
Ca%	0.66	0.75	2.39	1.4	1.88	2.96	2.58	2.18
P%	0.38	0.36	1.11	0.83	0.71	1.35	1.19	1.08

**Fig. 16:**
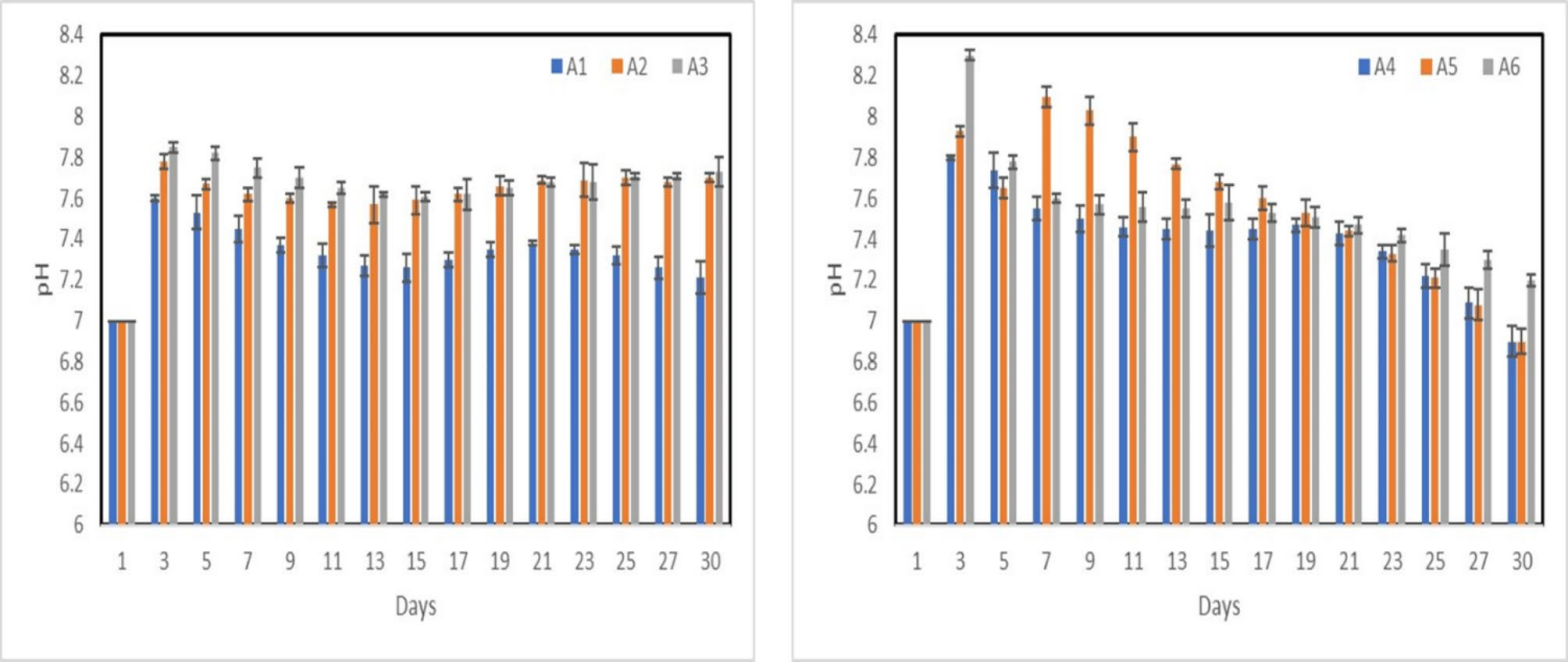
pH analysis of group A, PP-nHA (**a**) and PP-nHA-MWCNT (**b**).

**Fig. 17:**
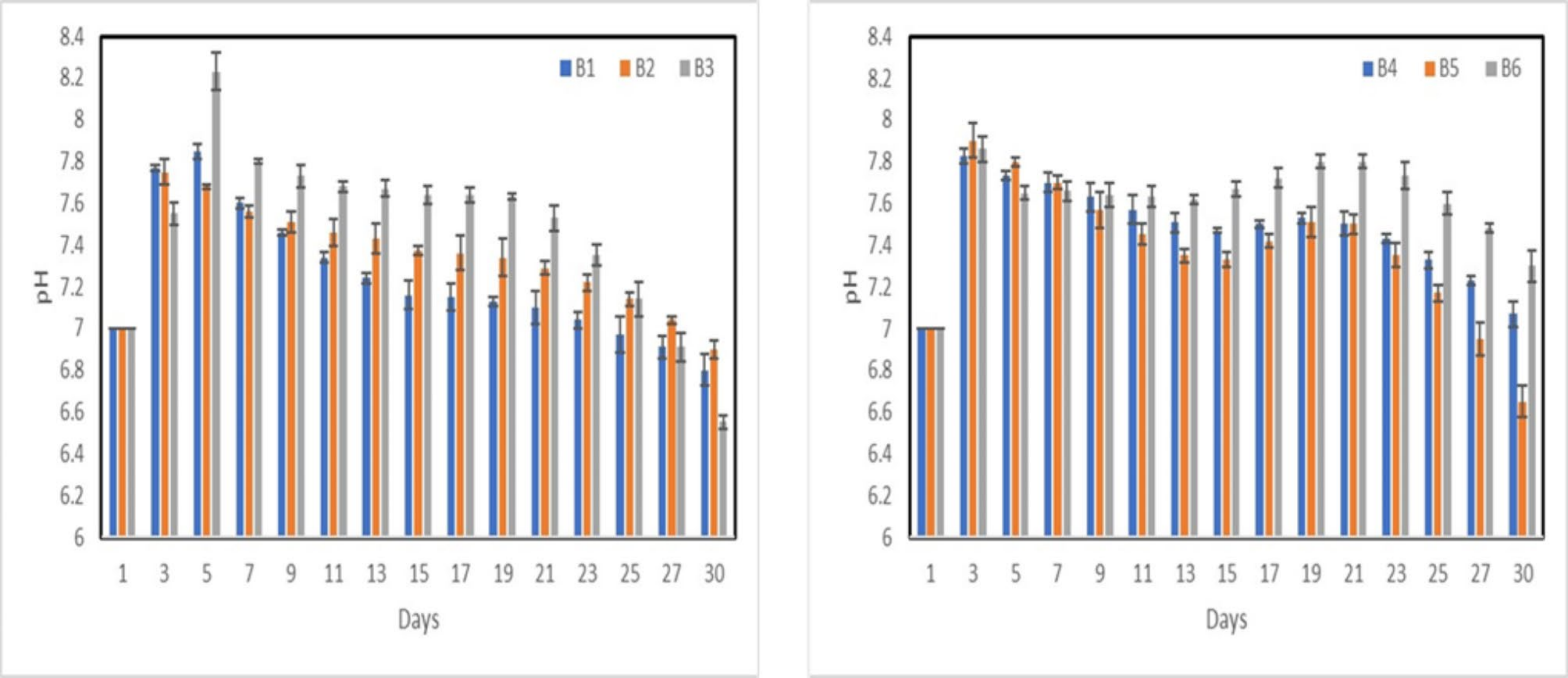
pH analysis of group B, PP-nHA (**a**) and PP-nHA-MWCNT (**b**).

PH analyses of nanocomposite for group A and B samples are represented in Figs. 16, 17 after immersion in SBF for different times. It was found that pH has increased at maximum of 8.3 after 3 days “sample A6” due to the fast release of alkali ions (Na^+^) and alkaline earth ions (Ca^2+^) and its exchange with H^+^ or H_3_O^+^ ions in the simulated body fluid (SBF) solution, thus increase the OH^−^ ions, which increase the pH of the solution. Soaking the samples in SBF led to the formation of an appetite layer on the surface of the composite, represented by the gradual decrease in the solution pH. Both nHA particles’ size and the MWCNTs don’t have a significant effect on the rate of the apatite precipitation on the composite surface.

## Conclusions

In the current investigation, PP based nano-composites reinforced with different sizes and contents of nHA particles and MWCNTs were successfully produced by melt blending. The following conclusions were driven;

- The incorporation of nHA particles and/or MWCNT into the polymer matrix didn’t cause any structural change to the PP crystalline lattice.
- The addition of MWCNTs caused a significant improvement in the dispersion of nHA particles along the PP matrix with a smoother surface pattern.
- The addition of MWCNTs and nHA exhibited a positive effect in enhancing the thermal strength of PP.
- Mechanical studies showed that the tensile strength of PP increased by 20% for PP-5 wt.%(90 nm) HA-MWCNTs composites and 44% for PP-5 wt.%(40 nm) HA-MWCNTs composites.
- The apatite forming ability was enhanced in the case of composite samples reinforced with both smaller sizes of nHA particles (40nm) and MWCNTs.
- PP based nanocomposites reinforced with 5 wt.% (40 nm HA particles) and 0.3 wt.% MWCNTs showed promising potential as bioabsorbable materials for osteofixation surgery.

## Electronic supplementary material

Below is the link to the electronic supplementary material.

Supplementary Material 1

## Data Availability

The authors declare that the data supporting the findings of this study are available within the paper.
