# Supplementary figures and images for: Development and characterization of hydroxyapatite and multiwall carbon nanotubes reinforced polypropylene biocomposites
Source: Sci Rep. 2025 May 28;15:18754. doi: 10.1038/s41598-025-96082-8 (PMC12119858; doi:10.1038/s41598-025-96082-8)

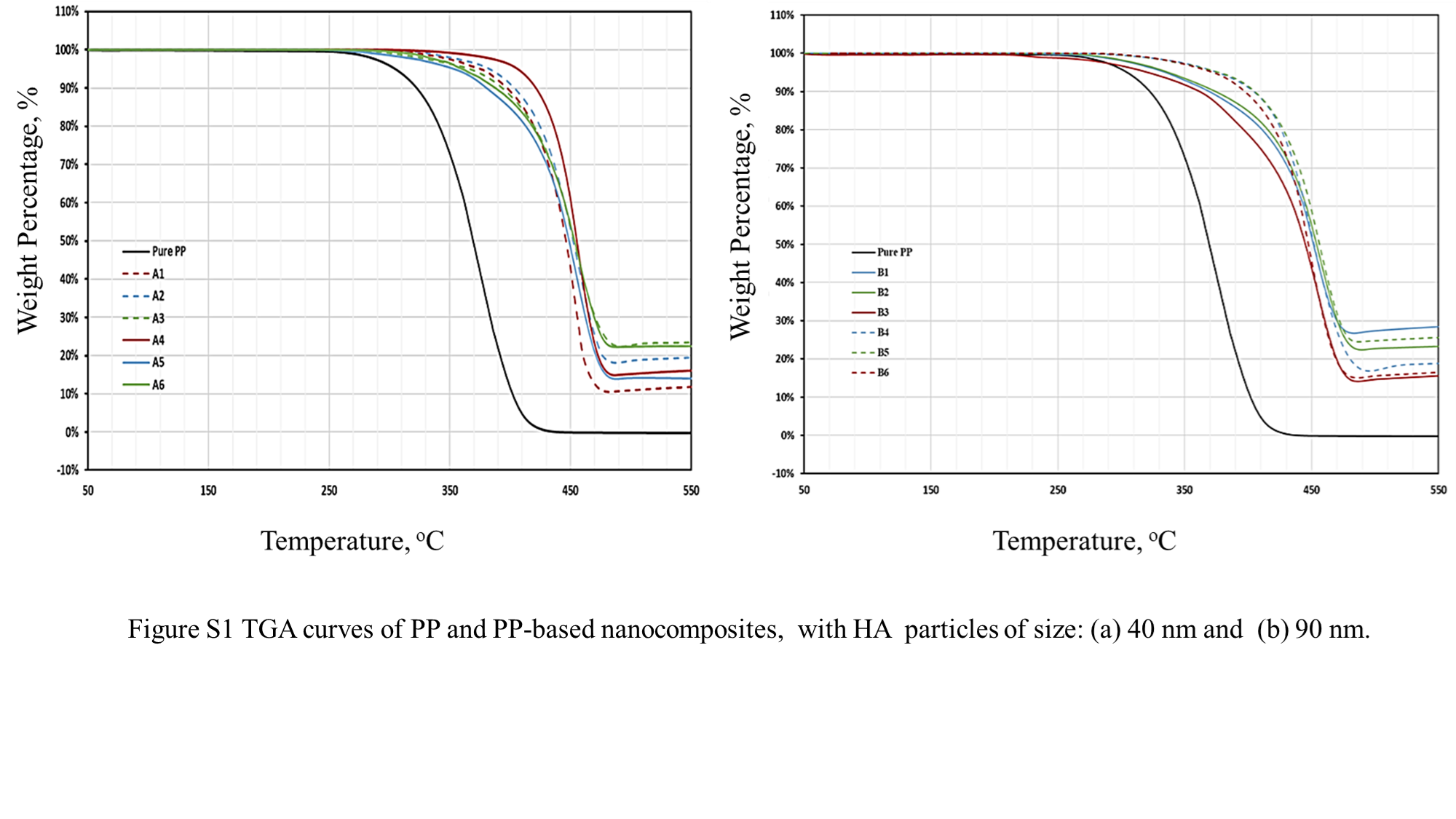

Supplement: Supplementary file 1 — Supplementary Material 1 [file 41598_2025_96082_MOESM1_ESM.tif]
